# Antitumor Efficacy and Mechanism in Hepatoma H22-Bearing Mice of *Brucea javanica* Oil

**DOI:** 10.1155/2015/217494

**Published:** 2015-10-05

**Authors:** Wen-Rong Shi, Yan Liu, Xiao-Ting Wang, Qiong-Ying Huang, Xue-Rong Cai, Shao-Rong Wu

**Affiliations:** College of Integrated Traditional Chinese and Western Medicine, Fujian University of Traditional Chinese Medicine, Fuzhou 350122, China

## Abstract

*Brucea javanica* is a traditional herbal medicine in China, and its antitumor activities are of research interest. *Brucea javanica* oil, extracted with ether and refined with 10% ethyl alcohol from *Brucea javanica* seed, was used to treat hepatoma H22-bearing mice in this study. The antitumor effect and probable mechanisms of the extracted *Brucea javanica* oil were studied in H22-bearing mice by WBC count, GOT, GPT levels, and western blotting. The H22 tumor inhibition ratio of 0.5, 1, and 1.5 g/kg bw *Brucea javanica* oil were 15.64%, 23.87%, and 38.27%. *Brucea javanica* oil could inhibit the involution of thymus induced by H22 tumor-bearing, but it could not inhibit the augmentation of spleen and liver. *Brucea javanica* oil could decrease the levels of WBC count and GOT and GPT in H22-bearing mice. The protein levels of GAPDH, Akt, TGF-*β*1, and *α*-SMA in tumor tissues decreased after being treated with *Brucea javanica* oil. Disturbing energy metabolism and neoplastic hyperplasia controlled by Akt and immunoregulation activity were its probable antitumor mechanisms in hepatoma H22-bearing mice.

## 1. Introduction

Cancer is a generic term for a large group of diseases that can affect any part of the body. One defining feature of cancer is the rapid creation of abnormal cells that grow beyond their usual boundaries and which can then invade adjoining parts of the body and spread to other organs. This process is referred to as metastasis. Metastases are the major cause of death from cancer. Cancer is a leading cause of death worldwide, accounting for 8.2 million deaths in 2012. The most common causes of cancer death are cancers of lung and liver [1]. Chinese medicine, one of the most popular complementary and alternative medicines, is an available option in many cancer centres in Asia, North America, and Europe [2].

*Brucea javanica* (*B. javanica *(L.) Merr.) is a shrub mostly originated in India, Southeast Asia, and Northern Australia [3].* Brucea javanica* seed is used for oncotherapy in Chinese medicine. A series of chemical compounds have been isolated from this plant, such as alkaloids, lignans, terpenoids, alkaloid glycosides, quassinoid glycosides, and quassinoids [4–8]. In addition, a complex mixture of fatty acids and fatty acid derivatives (*Brucea javanica* oil), whose main activity components are oleic acid and linoleic acid, has been extracted from the seed of* Brucea javanica.* Oleic acid, linoleic acid, and quassinoids are known to be the major antitumor activity compounds [9]. It has been reported that* Brucea javanica *oil inhibited tumor cell growth via inhibition of DNA polymerase, overcoming tumor multidrug resistance, and the damage of tumor cell membrane system [10].

*Brucea javanica* oil is a natural plant product that possesses antitumor properties, but more molecular mechanisms of the antitumor effects of it are still unrevealed. Here we investigate the antitumor efficacy of* Brucea javanica* oil extracted with ether and refined with 10% ethyl alcohol in hepatoma H22-bearing mice. Furthermore, the protein levels of *β*-catenin, PI3K, Akt, TGF-*β*1, *α*-SMA, GAPDH, and *β*-actin (as internal control) in H22 tumor tissues treated in vivo with* Brucea javanica* oil were detected.

## 2. Materials and Methods

### 2.1. Mice and Cell Lines

Female Chinese Kunming mice (weight 18~20 g) were purchased from Fujian medical university laboratory animal center (Fuzhou, China). The mice were housed under normal condition and with free access to food and water. Animal experiments and animal care were carried out according to protocols approved by the institutional committee for animal care and also in accordance with the policy of the National Ministry of Health. Murine hepatoma cell line H22 was purchased from China Centre for Type Culture Collection (CCTCC, Wuhan, China), subcultured, and maintained in our laboratory according to the guidelines given.

### 2.2. Extraction and Refining of* Brucea javanica* Oil

*Brucea javanica *fruits (Chinese medicinal materials, place of origin: Xiamen, China) were purchased from Suzhou Hengfeng Ginseng & Deer Antler Commercial Firm, Jiangsu, China.* Brucea javanica *fruits were dried to constant weight at 80°C and shelled to get the seeds.* Brucea javanica *seeds were milled and soaked with ether to extract the seed oil. The crude seed oil was refined with 10% ethyl alcohol according to the patented method [11].

### 2.3. Acute Toxicity in Mice

Kunming mice were randomly divided into 8 groups according to the dose (*n* = 12 each group). The refined* Brucea javanica *oil was injected subcutaneously in the back with doses of 1.25, 2.5, 5, 6.25, 7.5, 8.75, 10, and 11.25 g/kg bw. After 24 h of injection, the number of mice surviving was recorded and the value of LD_50_ of* Brucea javanica *oil was calculated using the Bliss method with BL-420E software (Chengdu TME Technology Co., Ltd., China) [12].

### 2.4. Antitumor Efficacy on Mice Hepatoma

H22-Bearing Mice models were generated by subcutaneous injection of 2 × 10^6^ H22 cells (mice hepatoma) in the armpit of left forelimb of each mouse. After injection, the mice models were randomly divided into 5 groups (*n* = 10 each group): soybean oil for injection (negative control), 0.5, 1, and 1.5 g/kg bw* Brucea javanica *oil (groups A, B, and C), and 25 mg/kg bw 5-Fu (positive control). And 10 other normal mice were set as normal control with injection of equal volume of soybean oil. The* Brucea javanica* oil was diluted to corresponding concentration with soybean oil for injection. The mice were injected subcutaneously in the back with corresponding medicines and the body weights before and after the experiment were measured. Seven days of continuous infusion later, all the mice were sacrificed, the tumor weights were recorded, and the whole blood, serum, thymus, spleen, heart, liver, kidney, and lung were collected. The blood white blood cells (WBC) were counted manually and the levels of GOT and GPT in serum were measured with automated biochemical analyzer (Hitachi, Japan). The organ coefficients (mg/g) of thymus, spleen, liver, kidney, and lung were calculated using the following formula: organ coefficient = organ weight/(body weight – tumor weight). Antitumor effects are expressed with inhibition ratio (%). The inhibition ratio (%) was calculated by the following formula: inhibition ratio (%) = [(*A* − *B*)/*A*] × 100%, where *A* is the average tumor weight of the negative control and *B* is the tumor weight of the treated group or positive control.

### 2.5. Antibodies for Western Blotting

Antibodies against *β*-catenin, Akt, and PI3K were from Cell Signaling Technology (Beverly, MA, USA). Antibodies against TGF-*β*1 and *α*-SMA were from Abcam (UK). The antibody against GAPDH was from Hangzhou Xianzhi Biological Technology (Zhejiang, CN). Anti-*β*-actin antibody was from Sigma. Anti-mouse IgG peroxidase-linked whole antibody and anti-rabbit IgG peroxidase-linked species-specific whole antibody were from Beyotime Institute of Biotechnology (Jiangsu, CN).

### 2.6. Tumor Harvesting and Western Blotting

The H22 tumors were excised from the mice of negative control group and 1.5 g/kg* Brucea javanica *oil groups and snap frozen in liquid nitrogen. Lysates were prepared in RIPA lysis buffer (Beyotime Institute of Biotechnology, Jiangsu, CN) using a dounce homogenizer. Protein concentrations were quantified using BCA Protein Assay Reagent (Beyotime Institute of Biotechnology, Jiangsu, CN). Lysates were run at 40 *μ*g per lane on 8% to 10% Bis-Tris gels and transferred to PVDF membranes (Invitrogen). Western blot band intensity quantification was done using Gel-Pro Analyzer software v4.0 (Media Cybernetics, Inc., USA). To account for differences in protein loading, all band intensities were corrected for *β*-actin.

### 2.7. Statistical Analysis

Student's *t*-test and ANOVA were used to analyze mean differences between groups of mice. *P* values of <0.05 were considered significant.

## 3. Results

### 3.1. Acute Toxicity in Mice

The LD_50_ was used to determine the acute toxicity, the changes in behavior, breathing, cutaneous effect, and sensory nervous system responses, and gastrointestinal effects were observed. The subcutaneous injection of* Brucea javanica *oil in doses ranging from 1.25 g/kg bw to 5 g/kg bw did not produce significant toxicity symptoms. Accompanying the increase of dose, towering hair, reduction in locomotor activity, and dull reactions were produced and the mortality was 100% in the dose of 11.25 g/kg bw. The LD_50_ of* Brucea javanica *oil and its 95% confidence limits were 8.36 and 7.07–10.05 g/kg bw.

### 3.2. Effect of* Brucea javanica *Oil on Body Weight of H22-Bearing Mice

The changes in body weights of mice before and after the experiment were as shown in Table 1; body weights increased markedly in all of the experimental groups. After experiment, the average body weights of mice did not have significant differences between six groups, although the average body weight of mice in positive control group was a little lower compared with normal control or negative control group. The subcutaneous injection of* Brucea javanica *oil for 7 days in dose no more than 1.5 g/kg bw did not affect the body weight growth.

### 3.3. Effect of* Brucea javanica *Oil on Tumor Weight and Tumor Inhibition Ratio

**Figure 1:**
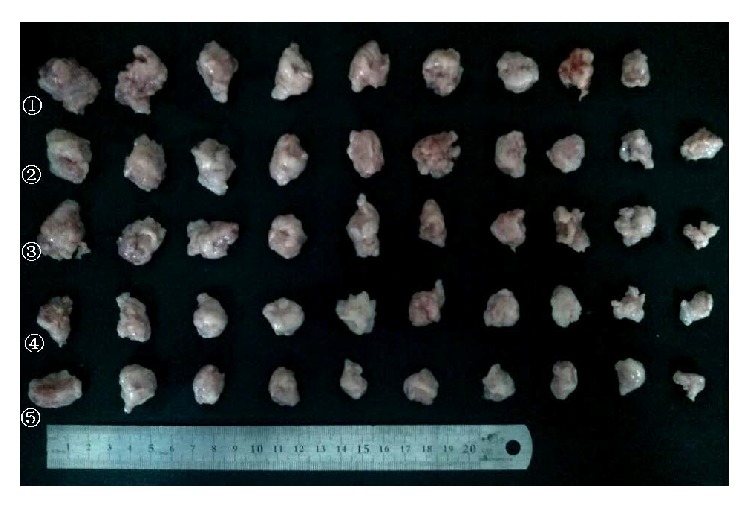
The tumors excised from the mice in the different groups. ① Negative group, ② 0.5 g/kg bw* Brucea javanica* oil, ③ 1 g/kg bw* Brucea javanica* oil, ④ 1.5 g/kg bw* Brucea javanica* oil, and ⑤ positive group (25 mg/kg bw 5-Fu).

The results for the effect of* Brucea javanica *oil on tumor weight and tumor inhibition ratio were as shown in Figure 1 and Table 2. There was a mouse in the negative control group that died in the sixth day, and only the body weight and tumor weight were recorded. The tumor excised from this mouse was not photographed.

### 3.4. Changes in Main Organ Coefficients of Mice

The main organ coefficients of mice, including thymus, spleen, liver, kidney, heart, and lung index, were as shown in Tables 3 and 4. Compared with the normal control group, H22 tumor-bearing decreased the thymus index and increased the spleen and liver indices significantly. Treated with 25 mg/kg bw 5-Fu, the thymus index of tumor-bearing mice decreased significantly. Compared with the negative control group, low dose* Brucea javanica* oil can increase the thymus index, but the high dose* Brucea javanica* oil decreased the thymus index instead (*P* = 0.08). The spleen index of tumor-bearing mice can be more or less increased by 0.5, 1, and 1.5 g/kg bw* Brucea javanica* oil. There were no significant differences in the indices of kidney, heart, and lung between all groups.

### 3.5. Effect of* Brucea javanica *Oil on WBC Count of Mice

The changes of average WBC count of mice were as shown in Table 5. It can be seen from the result H22 tumor-bearing increased WBC count of mice obviously and the WBC counts in 5-Fu treated or in* Brucea javanica *oil treated tumor-bearing mice decreased to the normal level.

### 3.6. Changes in GOT and GPT of Mice

The changes in the levels of GOT and GPT in serum of mice were as shown in Table 6. The results showed that Hepatoma H22-bearing increased the levels of GOT and GPT significantly and the levels of GOT and GPT decreased after being treated with 5-Fu or* Brucea javanica *oil compared with the negative control group.

### 3.7. Changes in Protein Levels of *β*-catenin, PI3K, Akt, GAPDH, *α*-SMA, and TGF-*β*1

**Figure 2:**
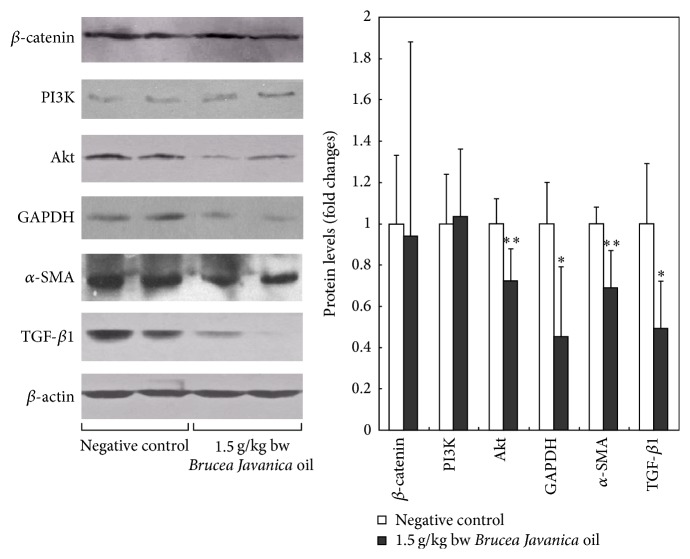
The protein levels of *β*-catenin, PI3K, Akt, GAPDH, *α*-SMA, and TGF-*β*1 in negative control group and 1.5 g/kg bw* Brucea javanica* oil group. The *β*-actin was as internal control. There were 8 samples detected randomly per group and the error bars correspond to mean ± standard deviations. Comparison with the negative control group, ^*∗*^ *P* < 0.05, ^*∗∗*^ *P* < 0.01.

The protein levels of *β*-catenin, PI3K, Akt, GAPDH, *α*-SMA, and TGF-*β*1 in negative control group and 1.5 g/kg bw* Brucea javanica *oil group were as shown in Figure 2.

## 4. Discussion

*Brucea javanica *fruit is a kind of Chinese herb with toxicity. In China, the emulsion formulation of* Brucea javanica *oil has been used clinically widely in combination with conventional therapy to treat carcinoma, demonstrating efficacy enhancing, toxicity reducing effects, and immunoregulation activity [13–16], but the exact antitumor active components and corresponding molecular mechanisms have not been fully clarified [17].

*Brucea javanica* oil has been found to exhibit lethal toxicity to human or experimental animals, which has brought many difficulties to clinical application. The water-soluble quassinoid compounds were considered as the major material basis of its toxicity such as brucenol, bruceoside, brusatol and bruceine [18, 19]. In our past study, the LD_50_ of* Brucea javanica* oil extracted with ether directly was 2.26 g/kg bw in mice. We used the 10% ethyl alcohol to extract and refine the* Brucea javanica* oil. After the removal of residual water-soluble toxic components, the LD_50_ of refined* Brucea javanica* oil increased to 8.36 g/kg bw in mice. Oleic acid and linoleic acid were considered as the major antitumor activity compounds in* Brucea javanica* oil [9]. In addition, three terpene alcohols, lupeol, and taraxerol also had antitumor activity [20–22]. Our results clearly indicated that the refined* Brucea javanica* oil can inhibit the growth of implanted hepatoma H22 in mice in a dose-dependent manner. The antitumor efficacy of high dose* Brucea javanica *oil (1.5 g/kg bw) was slightly lower than 25 mg/kg bw 5-Fu.

Then we investigate probable mechanisms of* Brucea javanica* oil in hepatoma H22-bearing mice. The multiple pathways involved in the action of* Brucea javanica* oil were further identified [17, 23–26]. It could be proposed* Brucea javanica* oil induces apoptotic death of cancer cells via both the death receptors and the mitochondrial-related pathways.* Brucea javanica* oil also could inhibit the invasion and migration of tumor cells targeting at MRP-1/CD9 and integrin alpha-5. In addition, the autophagic process contributed to an increasing rate of cell death induced by* Brucea javanica* oil. In this study, we concluded immunoregulation activity and neoplastic hyperplasia controlled by Akt were probable antitumor mechanisms of* Brucea javanica *oil in H22-bearing mice.

The thymus is the major site of T cell differentiation and a key organ of the immune system. Many studies proved that tumor-bearing in mice could induce thymic atrophy due to the abnormal T cell development and apoptosis. In tumor-bearing mice, T cell recruitment from the thymus to the spleen and splenic excess augmentation could be observed. The splenic excess augmentation in H22-bearing mice was associated with the export of cells which was restrained after the cells entered the spleen, especially the CD8+T cell being detected. CD8+T cell was bound up with immunosuppression of spleen in H22-bearing mice [27–30]. Our results showed that low and medium dose of* Brucea javanica *oil could prevent the thymic atrophy of mice induced with H22 tumor-bearing in different extent, but the high dose of* Brucea javanica *oil could not. Treated with high dose of* Brucea javanica *oil, the splenic excess augmentation in H22 tumor-bearing mice became serious. Whether the more serious splenic excess augmentation could cause more serious immunosuppression of spleen in H22-bearing mice or not needs further study.

According to the result of effect of* Brucea javanica *oil on WBC count of mice, it was showed that H22 tumor-bearing induced the increase of WBC count and 5-Fu as a myelosuppressive agent could decrease the WBC count of tumor-bearing mice.* Brucea javanica *oil could also decrease the WBC count of tumor-bearing mice to the normal level. The effect of* Brucea javanica *oil on inhibiting the increase of WBC count is not relevant to myelosuppressive effect perhaps due to its anti-inflammatory property [31].

Hepatocellular carcinoma cell line H22 can express hepatocyte growth factor (HGF), which is a potent stimulator of DNA synthesis in a variety of epithelial cells, including hepatocytes, and has been implicated in liver regeneration [32, 33]. This study showed that the liver index of mice increased significantly after hepatoma H22 was implanted in mice. Neither 5-Fu nor* Brucea javanica *oil could inhibit the liver augmentation induced with H22 tumor-bearing. It was reported that the levels of GOT and GPT in serum would increase significantly when H22 cells were implanted into mice, and cytoxan (CTX) could decrease the levels in H22 tumor-bearing mice [34]. Our results indicated that both 5-Fu and* Brucea javanica *oil could decrease the levels of GOT and GPT in H22 tumor-bearing mice in different extent and* Brucea javanica *oil in dose no more than 1.5 g/kg bw didnot have obvious toxicity to the liver.

Disturbing energy metabolism and neoplastic hyperplasia controlled by Akt could be another probable antitumor mechanisms in hepatoma H22-bearing mice. Akt is a serine/threonine protein kinase that plays an important role in cell growth, proliferation, and survival. Numerous studies have revealed the blockage of Akt signaling to result in apoptosis and growth inhibition of tumor cells [35]. Akt counteracts apoptosis through a block of caspase-9, phosphorylation of proapoptotic members of the Bcl2-family of mitochondria-targeting proteins such as BAD, and stimulating signaling pathway of NF-kB and is also involved in the regulation of autophagy [36], DNA damage response and repair induced by commonly used genotoxic agents [37], and normal vascularization and pathological angiogenesis. Therefore, this signaling pathway of downstream of Akt has been considered to be a new target for effective cancer therapeutic strategies. In this study, treated with* Brucea javanica *oil the level of Akt in tumor tissue decreased, suggesting the potential of triggering apoptosis of* Brucea javanica *oil in hepatoma H22 cells.

In contrast to normal differentiated cells, which rely primarily on mitochondrial oxidative phosphorylation to generate the energy needed for cellular processes, most cancer cells instead rely on aerobic glycolysis, a phenomenon termed “the Warburg effect.” GAPDH is an important enzyme for energy metabolism and the production of ATP and pyruvate through anaerobic glycolysis in the cytoplasm. Additionally, it participates in apoptosis, membrane trafficking, iron metabolism, nuclear activities, and receptor mediated cell signaling. It was reported that overexpressed GAPDH in tumor could bind to active Akt and limit its dephosphorylation. This would lead to Bcl-xL overexpression and escape from caspase-independent cell death [38]. Recent reports indicate that GAPDH has the ability to interact with Akt in many settings [39, 40]. Since most cancers have evolved multiple strategies such as hypoxia to evade programmed cell death, it is suggested that GAPDH-dependent Akt expression is protecting cancer cells from hypoxia. In this study, treated with* Brucea javanica *oil the level of GAPDH and Akt in tumor tissue decreased. It is indicated that* Brucea javanica *oil could inhibit the growth of implanted hepatoma H22 in mice by disturbing energy metabolism and neoplastic hyperplasia controlled by Akt.

The TGF-*β* and PI3K/Akt signaling pathways are used in cells to control numerous responses, including proliferation, apoptosis, and metastasis. TGF-*β* is known for its cytostatic effects in premalignant states and its prooncogenic activity in advanced cancers. But in other ways, TGF-*β*1 can stimulate tumor cells to produce growth factors and promote tumor growth and development. It can also induce epithelial-mesenchymal transition (EMT) and promote the invasion and metastasis of tumor. In addition, TGF-*β*1 can inhibit tumor immunity to promote tumor progression [41]. Although early studies suggested that these two pathways might counteract each other in balancing cell survival, emerging evidence has uncovered multiple modes of intricate signal integration and obligate collaboration in driving cancer progression [42]. In this study, it was showed that the protein level of TGF-*β*1 and Akt decreased after treating with* Brucea javanica *oil in H22 tumor tissues, suggesting the collaboration of the two signaling pathways.

Alpha-smooth muscle actin (*α*-SMA) is commonly used as a marker of myofibroblast formation and tumor microvascular density associates with *α*-SMA expression [43, 44]. Our results showed that* Brucea javanica *oil could inhibit the growth of H22 tumors with downregulation of *α*-SMA in tumor tissues. It implied that* Brucea javanica *oil could inhibit tumor angiogenesis probably to block the growth of H22 tumors.

As mentioned above, immunoregulation activity and neoplastic hyperplasia controlled by Akt were probable antitumor mechanisms of* Brucea javanica *oil in H22-bearing mice. The work on* Brucea javanica *oil's antitumor components and compatibility with other herbs to reduce acute toxicity and enhance its antitumor activity is underway now in our laboratory and will be communicated in due course.

## Figures and Tables

**Table 1:** Effect of *Brucea Javanica* oil on body weight of H22-bearing mice before and after experiment ($\overset{-}{X}\pm S$, *n* = 10).

Group	Dose (g/kg bw)	Number of animals	Average body weight of mice before the experiment (g)	Average body weight of mice after the experiment (g)
Normal control		10	19.32 ± 1.86	23.67 ± 2.08
Negative control		10	19.31 ± 1.63	22.35 ± 2.29
5-Fu	0.25	10	19.15 ± 1.49	21.14 ± 1.92
A	0.5	10	19.30 ± 1.73	23.49 ± 1.94
B	1	10	19.06 ± 1.67	22.35 ± 2.34
C	1.5	10	19.34 ± 1.79	22.39 ± 1.68

The average body weight of mice after the experiment excluding the tumor weight.

**Table 2:** Effect of *Brucea Javanica* oil on tumor weight and tumor inhibition ratio ($\overset{-}{X}\pm S$, *n* = 10).

Group	Dose (g/kg bw)	Number of animals	Average tumor weight (g)	Inhibition ratio (%)
Negative control		10	2.43 ± 1.08	—
5-Fu	0.025	10	1.40 ± 0.62^*∗∗*^	42.39
A	0.5	10	2.05 ± 0.78	15.64
B	1	10	1.85 ± 0.73	23.87
C	1.5	10	1.50 ± 0.39^*∗∗*^	38.27

Comparison with the negative control group, ^*∗∗*^ *P* < 0.01.

**Table 3:** Changes in thymus index, spleen index, and liver index of mice ($\overset{-}{X}\pm S$).

Group	Dose (g/kg bw)	Number of animals	Thymus index (mg/g)	Spleen index (mg/g)	Liver index (mg/g)
Normal control		10	5.01 ± 0.79	5.11 ± 1.35	46.79 ± 5.27
Negative control		9	2.72 ± 1.06^*∗∗*^	8.76 ± 2.11^*∗∗*^	66.01 ± 6.29^*∗∗*^
5-Fu	0.025	10	1.22 ± 0.32^*∗∗*,△△^	7.64 ± 1.77^*∗*^	62.33 ± 6.89^*∗∗*^
A	0.5	10	3.72 ± 1.28^*∗∗*,△^	10.83 ± 1.09^*∗∗*,△^	68.56 ± 6.76^*∗∗*^
B	1	10	3.17 ± 0.95^*∗∗*^	11.80 ± 3.09^*∗∗*,△△^	64.80 ± 6.72^*∗∗*^
C	1.5	10	2.00 ± 0.75^*∗∗*^	10.37 ± 3.08^*∗∗*^	64.28 ± 8.36^*∗∗*^

Comparison with the normal control group, ^*∗*^ *P* < 0.05,  ^*∗∗*^ *P* < 0.01. Comparison with the negative control group, ^△^ *P* < 0.05, ^△△^ *P* < 0.01.

**Table 4:** Changes in kidney index, heart index, and lung index of mice ($\overset{-}{X}\pm S$).

Group	Dose (g/kg bw)	Number of animals	Kidney index (mg/g)	Heart index (mg/g)	Lung index (mg/g)
Normal control		10	12.17 ± 0.84	5.29 ± 0.82	7.74 ± 2.46
Negative control		9	12.07 ± 0.49	5.26 ± 0.47	6.01 ± 0.48
5-Fu	0.025	10	12.88 ± 1.57	5.48 ± 0.69	6.41 ± 0.69
A	0.5	10	13.49 ± 2.52	5.87 ± 0.87	7.16 ± 1.26
B	1	10	13.08 ± 1.58	5.44 ± 0.87	7.20 ± 1.47
C	1.5	10	12.21 ± 1.99	4.83 ± 0.70	7.25 ± 2.34

**Table 5:** Changes of WBC count of mice ($\overset{-}{X}\pm S$).

Group	Dose (g/kg bw)	Number of animals	WBC count (10^9^ cells/L)
Normal control		10	4.79 ± 1.36
Negative control		9	13.88 ± 7.43^*∗∗*^
5-Fu	0.025	10	3.97 ± 1.22^△△^
A	0.5	10	5.25 ± 1.53^△△^
B	1	10	5.98 ± 2.36^△△^
C	1.5	10	5.58 ± 2.33^△△^

Comparison with the normal control group, ^*∗∗*^ *P* < 0.01; comparison with the negative control group, ^△△^ *P* < 0.01.

**Table 6:** Changes in the levels of GOT and GPT in serum of mice ($\overset{-}{X}\pm S$).

Group	Dose (g/kg bw)	Number of animals	GOT (U/L)	GPT (U/L)
Normal control		10	146.00 ± 33.93	70.04 ± 14.47
Negative control		9	780.16 ± 225.02^*∗∗*^	384.16 ± 70.41^*∗∗*^
5-Fu	0.025	10	395.00 ± 116.43^*∗∗*,△△^	172.67 ± 41.59^*∗∗*,△△^
A	0.5	10	589.60 ± 147.56^*∗∗*,△^	286.17 ± 71.84^*∗∗*,△△^
B	1	10	568.40 ± 248.91^*∗∗*,△^	276.60 ± 90.91^*∗∗*,△△^
C	1.5	10	665.66 ± 183.02^*∗∗*^	331.67 ± 91.01^*∗∗*^

Comparison with the normal control group, ^*∗∗*^ *P* < 0.01; comparison with the negative control group, ^△^ *P* < 0.05, ^△△^ *P* < 0.01.
